# Supplementary material for: Paving the way for human vaccination against Rift Valley fever virus: A systematic literature review of RVFV epidemiology from 1999 to 2021
Source: PLoS Negl Trop Dis. 2022 Jan 24;16(1):e0009852. doi: 10.1371/journal.pntd.0009852 (PMC8812886; doi:10.1371/journal.pntd.0009852)
Supplement: S1 Text — (DOCX) [file pntd.0009852.s001.docx]

**RVF Systematic Review information sources and online Search strategy**:

In order to maximize detection of eligible RVFV transmission studies, we searched the online databases PubMed (last searched 23 Nov 2020), Web of Science (searched 8 Dec 2020), African Journals Online (searched 15 Dec 2020), The Cumulative Index to Nursing and Allied Health Literature (CINAHL, searched 18 Dec 2020), The Scientific Electronic Library Online (SciELO, searched 15 Dec 2020), Elsevier ScienceDirect (searched 18 Dec 2020), ResearchGate, and the Program for Monitoring Emerging Diseases (ProMED) listserv site (<https://promedmail.org/promed-posts/>, searched 31 Jan 2021). Reports of human RVF outbreaks were checked against historical online listings of the World Health Organization (WHO) ([www.who.int/csr/don/archive/disease/rift_valley_fever/en/](http://www.who.int/csr/don/archive/disease/rift_valley_fever/en/)) and the US Center for Disease Control and Prevention (CDC) ([www.cdc.gov/vhf/rvf/outbreaks/summaries.html](http://www.cdc.gov/vhf/rvf/outbreaks/summaries.html)). Animal outbreak data recorded by the World Organization for Animal Health (OIE) was recovered from their databases 2005-2021 WAHIS (<https://wahis.oie.int/#/home>) and their older Handistatus II database (<https://web.oie.int//hs2/report.asp?lang=en>). Other sources of papers and reports were also retrieved included i) polling colleagues involved in RVF research or control for non-indexed ‘gray literature’, ii) using Google Scholar referrals for ‘similar papers’, iii) scanning of literature found in personal archives, and iv) obtaining non-indexed citations found among the reference lists of the papers reviewed in our study. Unpublished data from a recent survey study in Nigeria was also included.

**For PubMed—**

(Rift Valley Fever) AND (incidence OR prevalence OR sero* OR epidemiology OR transmission OR disease outbreaks OR mortality OR complications OR risk assessment OR risk factors OR forecasting OR genetics OR strain OR lineage OR disease reservoir OR floods OR El Nino OR surveillance OR socioeconomic factors OR occupational diseases OR pastoralists OR transients OR migrants)

Time limit 1999-present

Search: (Rift Valley Fever) AND (incidence OR prevalence OR sero* OR epidemiology OR transmission OR disease outbreaks OR mortality OR complications OR risk assessment OR risk factors OR forecasting OR genetics OR strain OR lineage OR disease reservoir OR floods OR El Nino OR surveillance OR socioeconomic factors OR occupational diseases OR pastoralists OR transients OR migrants) Filters: from 1999 - 2021 Sort by: Most Recent

("rift valley fever"[MeSH Terms] OR ("rift"[All Fields] AND "valley"[All Fields] AND "fever"[All Fields]) OR "rift valley fever"[All Fields]) AND ("epidemiology"[MeSH Subheading] OR "epidemiology"[All Fields] OR "incidence"[All Fields] OR "incidence"[MeSH Terms] OR "incidences"[All Fields] OR "incident"[All Fields] OR "incidents"[All Fields] OR ("epidemiology"[MeSH Subheading] OR "epidemiology"[All Fields] OR "prevalence"[All Fields] OR "prevalence"[MeSH Terms] OR "prevalance"[All Fields] OR "prevalences"[All Fields] OR "prevalence s"[All Fields] OR "prevalent"[All Fields] OR "prevalently"[All Fields] OR "prevalents"[All Fields]) OR "sero*"[All Fields] OR ("epidemiologies"[All Fields] OR "epidemiology"[MeSH Subheading] OR "epidemiology"[All Fields] OR "epidemiology"[MeSH Terms] OR "epidemiology s"[All Fields]) OR ("transmissability"[All Fields] OR "transmissable"[All Fields] OR "transmissibilities"[All Fields] OR "transmissibility"[All Fields] OR "transmissible"[All Fields] OR "transmissibles"[All Fields] OR "transmission"[MeSH Subheading] OR "transmission"[All Fields] OR "transmissions"[All Fields]) OR ("disease outbreaks"[MeSH Terms] OR ("disease"[All Fields] AND "outbreaks"[All Fields]) OR "disease outbreaks"[All Fields]) OR ("mortality"[MeSH Terms] OR "mortality"[All Fields] OR "mortalities"[All Fields] OR "mortality"[MeSH Subheading]) OR ("complicances"[All Fields] OR "complicate"[All Fields] OR "complicated"[All Fields] OR "complicates"[All Fields] OR "complicating"[All Fields] OR "complication"[All Fields] OR "complication s"[All Fields] OR "complications"[MeSH Subheading] OR "complications"[All Fields]) OR ("risk assessment"[MeSH Terms] OR ("risk"[All Fields] AND "assessment"[All Fields]) OR "risk assessment"[All Fields]) OR ("risk factors"[MeSH Terms] OR ("risk"[All Fields] AND "factors"[All Fields]) OR "risk factors"[All Fields]) OR ("forecasted"[All Fields] OR "forecaster"[All Fields] OR "forecasters"[All Fields] OR "forecasting"[MeSH Terms] OR "forecasting"[All Fields] OR "forecast"[All Fields] OR "forecasts"[All Fields] OR "trends"[MeSH Subheading] OR "trends"[All Fields]) OR ("genetic therapy"[MeSH Terms] OR ("genetic"[All Fields] AND "therapy"[All Fields]) OR "genetic therapy"[All Fields] OR "genetic"[All Fields] OR "genetical"[All Fields] OR "genetically"[All Fields] OR "genetics"[MeSH Subheading] OR "genetics"[All Fields] OR "genetics"[MeSH Terms]) OR ("sprains and strains"[MeSH Terms] OR ("sprains"[All Fields] AND "strains"[All Fields]) OR "sprains and strains"[All Fields] OR "strain"[All Fields] OR "strains"[All Fields] OR "strain s"[All Fields]) OR ("lineage"[All Fields] OR "lineage s"[All Fields] OR "lineages"[All Fields]) OR ("disease reservoirs"[MeSH Terms] OR ("disease"[All Fields] AND "reservoirs"[All Fields]) OR "disease reservoirs"[All Fields] OR ("disease"[All Fields] AND "reservoir"[All Fields]) OR "disease reservoir"[All Fields]) OR ("floodings"[All Fields] OR "floods"[MeSH Terms] OR "floods"[All Fields] OR "flood"[All Fields] OR "flooded"[All Fields] OR "implosive therapy"[MeSH Terms] OR ("implosive"[All Fields] AND "therapy"[All Fields]) OR "implosive therapy"[All Fields] OR "flooding"[All Fields]) OR ("el nino southern oscillation"[MeSH Terms] OR ("el"[All Fields] AND "nino southern"[All Fields] AND "oscillation"[All Fields]) OR "el nino southern oscillation"[All Fields] OR ("el"[All Fields] AND "nino"[All Fields]) OR "el nino"[All Fields]) OR ("epidemiology"[MeSH Subheading] OR "epidemiology"[All Fields] OR "surveillance"[All Fields] OR "epidemiology"[MeSH Terms] OR "surveilance"[All Fields] OR "surveillances"[All Fields] OR "surveilled"[All Fields] OR "surveillence"[All Fields]) OR ("socioeconomic factors"[MeSH Terms] OR ("socioeconomic"[All Fields] AND "factors"[All Fields]) OR "socioeconomic factors"[All Fields]) OR ("occupational diseases"[MeSH Terms] OR ("occupational"[All Fields] AND "diseases"[All Fields]) OR "occupational diseases"[All Fields]) OR ("pastoralist"[All Fields] OR "pastoralists"[All Fields]) OR ("transiently"[All Fields] OR "transients and migrants"[MeSH Terms] OR ("transients"[All Fields] AND "migrants"[All Fields]) OR "transients and migrants"[All Fields] OR "transient"[All Fields] OR "transients"[All Fields]) OR ("migrant s"[All Fields] OR "transients and migrants"[MeSH Terms] OR ("transients"[All Fields] AND "migrants"[All Fields]) OR "transients and migrants"[All Fields] OR "migrant"[All Fields] OR "migrants"[All Fields]))

**Translations**

Rift Valley Fever: "rift valley fever"[MeSH Terms] OR ("rift"[All Fields] AND "valley"[All Fields] AND "fever"[All Fields]) OR "rift valley fever"[All Fields]

incidence: "epidemiology"[Subheading] OR "epidemiology"[All Fields] OR "incidence"[All Fields] OR "incidence"[MeSH Terms] OR "incidences"[All Fields] OR "incident"[All Fields] OR "incidents"[All Fields]

prevalence: "epidemiology"[Subheading] OR "epidemiology"[All Fields] OR "prevalence"[All Fields] OR "prevalence"[MeSH Terms] OR "prevalance"[All Fields] OR "prevalences"[All Fields] OR "prevalence's"[All Fields] OR "prevalent"[All Fields] OR "prevalently"[All Fields] OR "prevalents"[All Fields]

epidemiology: "epidemiologies"[All Fields] OR "epidemiology"[Subheading] OR "epidemiology"[All Fields] OR "epidemiology"[MeSH Terms] OR "epidemiology's"[All Fields]

transmission: "transmissability"[All Fields] OR "transmissable"[All Fields] OR "transmissibilities"[All Fields] OR "transmissibility"[All Fields] OR "transmissible"[All Fields] OR "transmissibles"[All Fields] OR "transmission"[Subheading] OR "transmission"[All Fields] OR "transmissions"[All Fields]

disease outbreaks: "disease outbreaks"[MeSH Terms] OR ("disease"[All Fields] AND "outbreaks"[All Fields]) OR "disease outbreaks"[All Fields]

mortality: "mortality"[MeSH Terms] OR "mortality"[All Fields] OR "mortalities"[All Fields] OR "mortality"[Subheading]

complications: "complicances"[All Fields] OR "complicate"[All Fields] OR "complicated"[All Fields] OR "complicates"[All Fields] OR "complicating"[All Fields] OR "complication"[All Fields] OR "complication's"[All Fields] OR "complications"[Subheading] OR "complications"[All Fields]

risk assessment: "risk assessment"[MeSH Terms] OR ("risk"[All Fields] AND "assessment"[All Fields]) OR "risk assessment"[All Fields]

risk factors: "risk factors"[MeSH Terms] OR ("risk"[All Fields] AND "factors"[All Fields]) OR "risk factors"[All Fields]

forecasting: "forecasted"[All Fields] OR "forecaster"[All Fields] OR "forecasters"[All Fields] OR "forecasting"[MeSH Terms] OR "forecasting"[All Fields] OR "forecast"[All Fields] OR "forecasts"[All Fields] OR "trends"[Subheading] OR "trends"[All Fields]

genetics: "genetic therapy"[MeSH Terms] OR ("genetic"[All Fields] AND "therapy"[All Fields]) OR "genetic therapy"[All Fields] OR "genetic"[All Fields] OR "genetical"[All Fields] OR "genetically"[All Fields] OR "genetics"[Subheading] OR "genetics"[All Fields] OR "genetics"[MeSH Terms]

strain: "sprains and strains"[MeSH Terms] OR ("sprains"[All Fields] AND "strains"[All Fields]) OR "sprains and strains"[All Fields] OR "strain"[All Fields] OR "strains"[All Fields] OR "strain's"[All Fields]

lineage: "lineage"[All Fields] OR "lineage's"[All Fields] OR "lineages"[All Fields]

disease reservoir: "disease reservoirs"[MeSH Terms] OR ("disease"[All Fields] AND "reservoirs"[All Fields]) OR "disease reservoirs"[All Fields] OR ("disease"[All Fields] AND "reservoir"[All Fields]) OR "disease reservoir"[All Fields]

floods: "floodings"[All Fields] OR "floods"[MeSH Terms] OR "floods"[All Fields] OR "flood"[All Fields] OR "flooded"[All Fields] OR "implosive therapy"[MeSH Terms] OR ("implosive"[All Fields] AND "therapy"[All Fields]) OR "implosive therapy"[All Fields] OR "flooding"[All Fields]

El Nino: "el nino-southern oscillation"[MeSH Terms] OR ("el"[All Fields] AND "nino-southern"[All Fields] AND "oscillation"[All Fields]) OR "el nino-southern oscillation"[All Fields] OR ("el"[All Fields] AND "nino"[All Fields]) OR "el nino"[All Fields]

surveillance: "epidemiology"[Subheading] OR "epidemiology"[All Fields] OR "surveillance"[All Fields] OR "epidemiology"[MeSH Terms] OR "surveilance"[All Fields] OR "surveillances"[All Fields] OR "surveilled"[All Fields] OR "surveillence"[All Fields]

socioeconomic factors: "socioeconomic factors"[MeSH Terms] OR ("socioeconomic"[All Fields] AND "factors"[All Fields]) OR "socioeconomic factors"[All Fields]

occupational diseases: "occupational diseases"[MeSH Terms] OR ("occupational"[All Fields] AND "diseases"[All Fields]) OR "occupational diseases"[All Fields]

pastoralists: "pastoralist"[All Fields] OR "pastoralists"[All Fields]

transients: "transiently"[All Fields] OR "transients and migrants"[MeSH Terms] OR ("transients"[All Fields] AND "migrants"[All Fields]) OR "transients and migrants"[All Fields] OR "transient"[All Fields] OR "transients"[All Fields]

migrants: "migrant's"[All Fields] OR "transients and migrants"[MeSH Terms] OR ("transients"[All Fields] AND "migrants"[All Fields]) OR "transients and migrants"[All Fields] OR "migrant"[All Fields] OR "migrants"[All Fields]

**For Web of Science—**

(ALL=Rift Valley Fever) AND ((ALL=incidence) OR (ALL=prevalence) OR (ALL=sero*) OR (ALL=epidemiology) OR (ALL=transmission) OR (ALL=disease outbreaks) OR (ALL=mortality) OR (ALL=complications) OR (ALL=risk assessment) OR (ALL=risk factors) OR (ALL=forecasting) OR (ALL=genetics) OR (ALL=strain) OR (ALL=lineage) OR (ALL=disease reservoir) OR (ALL=floods) OR (ALL=El Nino) OR (ALL=surveillance) OR (ALL=socioeconomic factors) OR (ALL=occupational diseases) OR (ALL=pastoralists) OR (ALL=transients) OR (ALL=migrants))

Indexes=SCI-EXPANDED, SSCI, A&HCI, CPCI-S, CPCI-SSH, BKCI-S, BKCI-SSH, ESCI, CCR-EXPANDED, IC Timespan=1999-2020

**For SciELO—**

Rift Valley Fever [All indexes] and incidence [All indexes] or prevalence [All indexes] or sero* [All indexes] or epidemiology [All indexes] or transmission [All indexes] or [All indexes] disease outbreaks [All indexes] or mortality [All indexes] or complications [All indexes] or risk assessment [All indexes] or risk factors [All indexes] or forecasting [All indexes] or genetics [All indexes] or strain [All indexes] or lineage [All indexes] or disease reservoir [All indexes] or floods [All indexes] or El Nino [All indexes] or surveillance [All indexes] or socioeconomic factors [All indexes] or occupational diseases [All indexes] or pastoralists [All indexes] or transients [All indexes] or migrants[All indexes] and 1999 [Publication year] or 2000 [Publication year or 2001 [Publication year] or 2002 [Publication year] or 2003 [Publication year] or 2004 [Publication year] or 2005 [Publication year] or 2006 [Publication year] or 2007 [Publication year] or 2008 [Publication year] or 2009 [Publication year] or 2010 [Publication year] or 2011 [Publication year] or 2012 [Publication year] or 2013 [Publication year] or 2014 [Publication year] or 2015 [Publication year] or 2016 [Publication year] or 2017 [Publication year] or 2018 [Publication year] or 2019 [Publication year] or 2020 [Publication year]

**For Africa Journals Online—**

"Rift Valley fever" AND " incidence" OR OR OR " prevalence" OR OR OR "sero*" OR OR OR "epidemiology" OR OR OR “transmission” OR OR OR "outbreak" OR OR OR “mortality” OR OR OR "complications" OR OR OR "risk assessment" OR OR OR "risk factor" OR OR OR "forecasting” OR OR OR “genetics” OR OR OR "strain" OR OR OR "lineage" OR OR OR "disease reservoir" OR OR OR "flood" OR OR OR "El Nino" OR OR OR "surveillance" OR OR OR "economic” OR OR OR “occupation” OR OR OR “pastoralist” OR OR OR “migrant”

**For CINAHL—**

“Rift Valley Fever” and 1999-2020

**For Elsevier ScienceDirect—**

“Rift Valley fever” restricted to 1999-2021. Not chapter; not review; not encyclopedia; “Rift” in title, abstract or key words;
