# Supplementary figures and images for: Paving the way for human vaccination against Rift Valley fever virus: A systematic literature review of RVFV epidemiology from 1999 to 2021
Source: PLoS Negl Trop Dis. 2022 Jan 24;16(1):e0009852. doi: 10.1371/journal.pntd.0009852 (PMC8812886; doi:10.1371/journal.pntd.0009852)

# Odds Ratio of RVFV Infection via Butchering

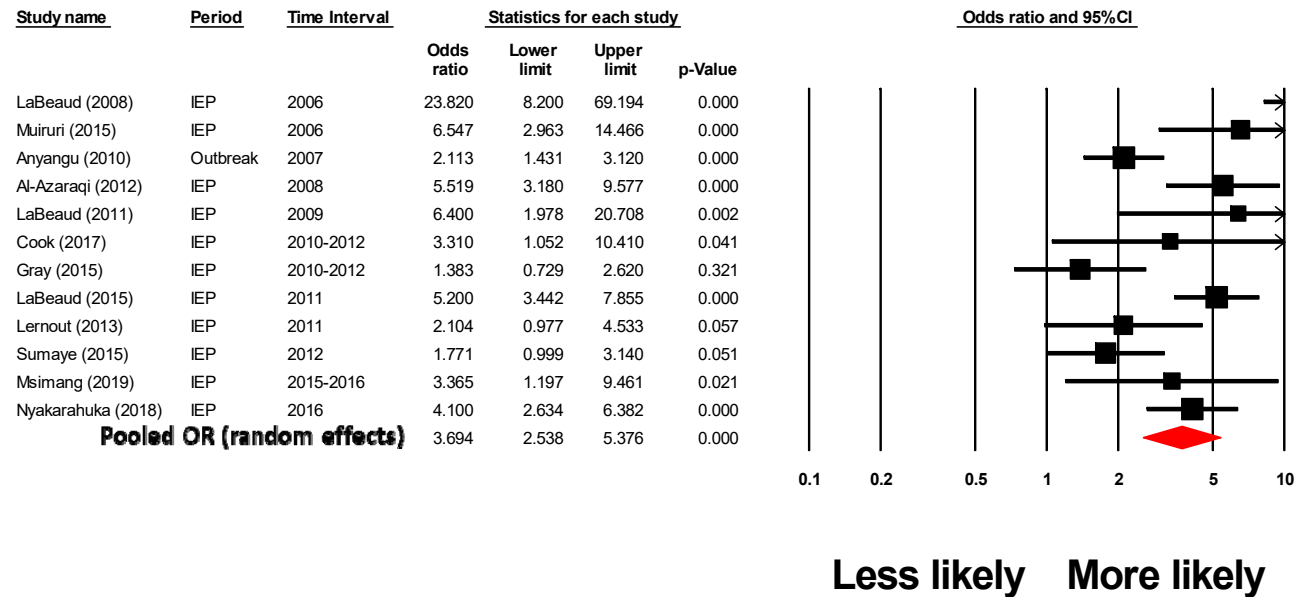

Supplement: S1 Fig — (PDF) [file pntd.0009852.s002.pdf]

# Odds Ratio of RVFV Infection via Sheltering Livestock

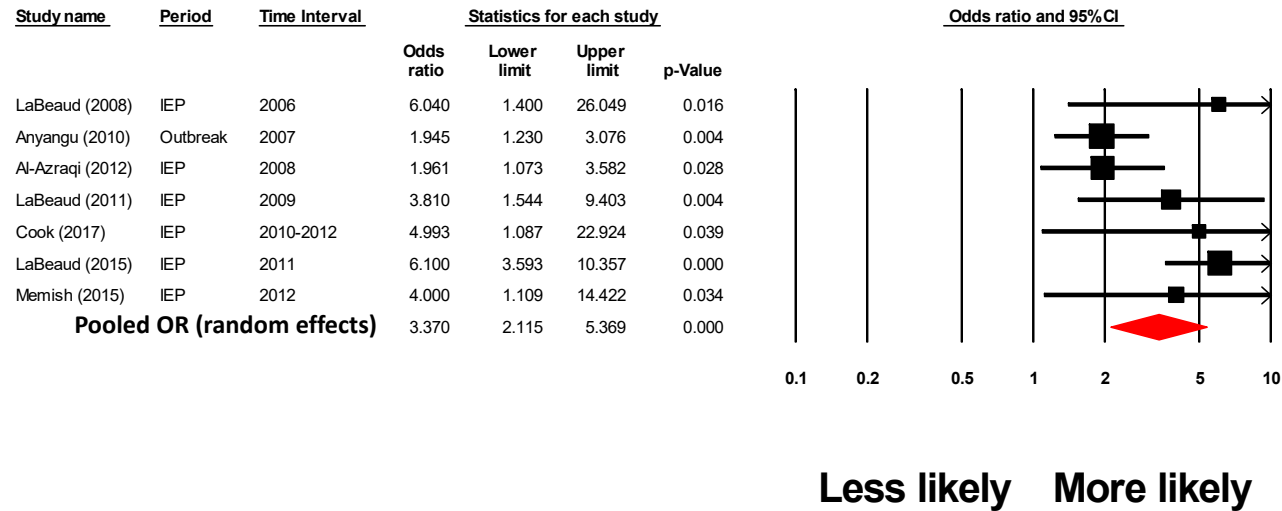

Supplement: S2 Fig — (PDF) [file pntd.0009852.s003.pdf]

# Odds Ratio of RVFV Infection via Milking Livestock

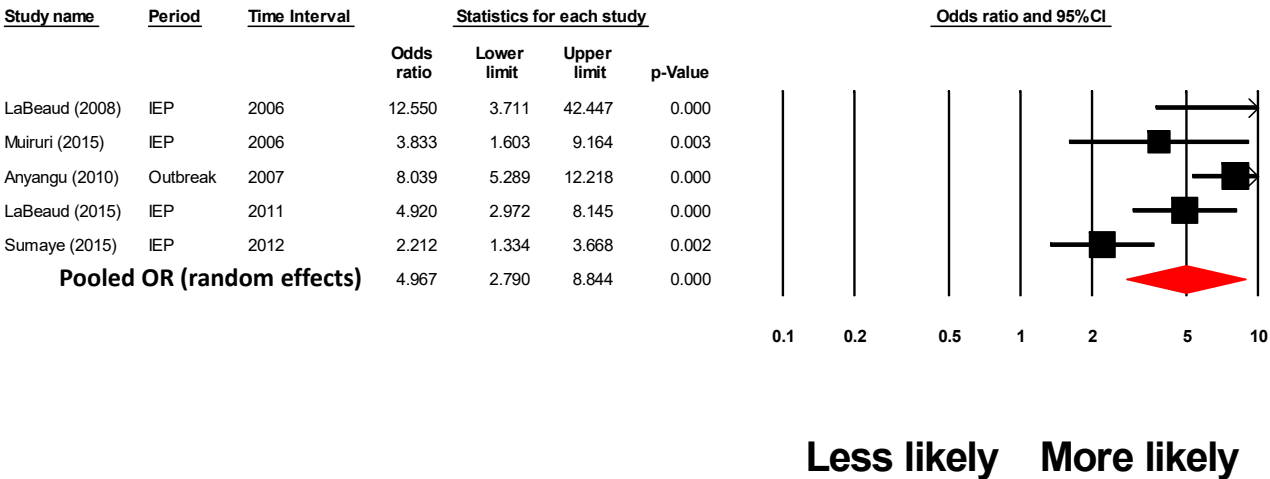

Supplement: S3 Fig — (PDF) [file pntd.0009852.s004.pdf]
